# Electroacupuncture and human iPSC-derived small extracellular vesicles regulate the gut microbiota in ischemic stroke *via* the brain-gut axis

**DOI:** 10.3389/fimmu.2023.1107559

**Published:** 2023-01-20

**Authors:** Qiongqiong Zhang, Peiying Deng, Suhui Chen, Hong Xu, Yamin Zhang, Hui Chen, Jianmin Zhang, Hua Sun

**Affiliations:** ^1^ Department of Traditional Chinese Medicine, Peking Union Medical College Hospital, Peking Union Medical College, Chinese Academy of Medical Sciences, Beijing, China; ^2^ CAMS Key Laboratory for T-Cell and Immunotherapy, State Key Laboratory of Medical Molecular Biology, Department of Immunology, Institute of Basic Medical Sciences, Chinese Academy of Medical Sciences and School of Basic Medicine, Peking Union Medical College, Beijing, China; ^3^ Haihe Laboratory of Cell Ecosystem, Chinese Academy of Medical Sciences and Peking Union Medical College, Tianjin, China; ^4^ Changzhou Xitaihu Institute for Frontier Technology of Cell Therapy, Changzhou, China; ^5^ Guidon Pharmaceutics, Inc., Beijing, China

**Keywords:** electroacupuncture, ischemic stroke, induced pluripotent stem cell, microbiome-gut-brain axis, inflammation

## Abstract

Electroacupuncture (EA) and induced pluripotent stem cell (iPSC)-derived small extracellular vesicles (iPSC-EVs) have substantial beneficial effects on ischemic stroke. However, the detailed mechanisms remain unclear. Here, we explored the mechanisms underlying the regulation of EA and iPSC-EVs in the microbiome-gut-brain axis (MGBA) after ischemic stroke. Ischemic stroke mice (C57BL/6) were subjected to middle cerebral artery occlusion (MCAO) or Sham surgery. EA and iPSC-EVs treatments significantly improved neurological function and neuronal and intestinal tract injury, downregulated the levels of IL-17 expression and upregulated IL-10 levels in brain and colon tissue after cerebral ischemia−reperfusion. EA and iPSC-EVs treatments also modulated the microbiota composition and diversity as well as the differential distribution of species in the intestines of the mice after cerebral ischemia−reperfusion. Our results demonstrated that EA and iPSC-EVs treatments regulated intestinal immunity through MGBA regulation of intestinal microbes, reducing brain and colon damage following cerebral ischemia and positively impacting the outcomes of ischemic stroke. Our findings provide new insights into the application of EA combined with iPSC-EVs as a treatment for ischemic stroke.

## 1 Introduction

Stroke seriously endangers human health and quality of life (1). Every 6 seconds, one person in the world dies of stroke (2, 3), and 60%-80% of these cases are due to ischemic stroke (4). Recent studies have demonstrated that stroke has a strong inflammatory response, intestinal symbiotic bacteria regulate ischemic stroke injury, and there is two-way communication between the brain and the gut called the “Microbiome-Gut-Brain Axis” (MGBA) (5–7).

The interaction of the components of this axis affects the occurrence, development, and prognosis of many neurological diseases (8–10). After stroke, communication from the brain to the intestine *via* the gut-brain axis may occur *via* parasympathetic and sympathetic efferent fibers that innervate the intestine directly or indirectly through the enteric nervous system, affecting the level of intestinal mucosal tight junctions and maintaining the integrity of the intestinal mucosal barrier and the gut microbiota (11). The gut microbiota and its release of neurotransmitters, metabolites, and immune factors affect the central nervous system and brain function after stroke by affecting immune cell differentiation and accumulation and changing the levels of circulating anti-inflammatory and proinflammatory cytokines (12). Modulation of the gut microbiome has the potential to prevent and treat stroke (13).

Immune signals can be exchanged between the brain after stroke and the gut following gut microbiota dysbiosis (GMD) (14, 15). After cerebral ischemia, the intestinal microbiota further damages the immune system, regulates lymphocytes, affects Treg cells and γδT cells, promotes IL-17 secretion, and aggravates intestinal and brain injury (16, 17). Treg cells play a neuroprotective role by secreting IL-10 to inhibit postischemic inflammation (18). Knockdown of IL-17 alleviates cerebral injury and restores the homeostasis of intestinal flora (19, 20). Transplantation of healthy intestinal flora (21, 22) and administration of exogenous compounds, such as short-chain fatty acids and fructose, induced the differentiation of T lymphocytes into Th1 cells and the secretion of IL-10 to eliminate inflammation (23, 24). IL-10 has a critical function in limiting the signaling of the proinflammatory factor IL-17A during stroke (25). The intestinal microbiota, IL-10, and IL-17A are closely related to recovery from cerebral injury (26, 27).

Induced pluripotent stem cell-derived small extracellular vesicles (iPSC-EVs) had therapeutic potential during stroke (28). iPSC-EVs could cross the blood−brain barrier and exhibit protective function against cerebral injury induced by ischemia−reperfusion (29, 30). Recent studies showed that exosomes derived from iPSCs or MSCs reduced infarct volume and brain edema, inhibited inflammation and promoted white matter integrity and functional recovery (31–34). Exosomes released by cells of the intestinal mucosa could modulate intestinal homeostasis, including epithelial barrier integrity, and orchestrate the regulation of intestinal immunity as well as the features of the microbiota (35, 36).

Electroacupuncture (EA), an effective acupuncture method, has been widely used for the treatment of stroke in the clinic. After acupuncture is used to get qi at the acupoint, a trace current wave of human bioelectricity is passed on the needle, which can objectively control the amount of stimulation. EA can reduce brain edema, inhibit inflammation and improve neurological function (37, 38). Acupuncture at “Baihui” (GV20) and “Zusanli” (ST36) and iPSC-EVs treatment upregulated Treg cells, suppressed Th17 and Th1-cell responses and exerted neuroprotective effects in an ischemia−reperfusion mouse model (29). However, the specific mechanism by which EA and iPSC-EVs regulate neuroinflammation and the gut microbiota remains unclear.

Accordingly, in this study, we explored the effects of EA treatment and iPSC-EVs in middle cerebral artery occlusion (MCAO) mice and found that EA and iPSC-EVs regulated the inflammation and gut microbiota response.

## 2 Materials and methods

### 2.1 Animals

Male mice (C57BL/6) aged 6~8 weeks and weighing 21~23 g (n= 10/group) were purchased from Beijing SPF Laboratory Animal Technology Co., Ltd. (Beijing, China). The mice were housed in a 12 h light/dark cycle in a temperature-controlled room (22 ± 2°C) and provided access to food and water. The animal treatment protocols were conducted according to the guidelines of the National Institutes for Animal Research and approved by the Ethics Committee for Animal Experimentation of Peking Union Medical College Hospital of the Chinese Academy of Medical Sciences (reference no. XHDW-2021-037).

### 2.2 Mouse model of middle cerebral artery occlusion

A mouse model of MCAO was established as previously described (29). The MCAO mice were successfully established by inserting a 7-0 monofilament (Doccol Corporation, Sharon, MA, USA) *via* the right external carotid artery (ECA) across the right internal carotid artery (ICA) to the origin of the middle cerebral artery (MCA) 1 h after withdrawing the monofilament.

MCAO in mice was established using a laser speckle contrast imager (PeriCam PSI HR System, Jarfalla-Stockholm, Sweden) while supervising regional cerebral blood flow (rCBF). Animals were excluded that died or failed to show a ≤50% or ≥80% rCBF reduction for further analysis (39–41). Sham group mice underwent the same procedure but without MCAO. After the operation, the mice were housed individually and euthanized by cervical dislocation after 72 h, and brain tissue, colon tissue and colon contents were collected for further experiments. The model success rate was 87.72%.

### 2.3 The preparation of iPSC-EVs

The iPS cells were amplified and cultured to logarithmic growth stage and were passaged at a ratio of 1:4~1:6 every 4~5 days as described previously (29). In brief, cell coverage reached 60-70% after adherent, and then the cells were cultured overnight. The culture medium was changed to GDEV medium for 24h, and the supernatant of cell culture was collected when the cell coverage reached 80-90%. The collected cell supernatant was ultrafiltrated to harvest exosomes. The purified exosomes were stored in a -80°C refrigerator for subsequent experiments.

### 2.4 EA and iPSC-EVs treatments

The mice were randomly divided into five groups, including the sham operation group (Sham), model group (MCAO), electroacupuncture group (EA), iPSC-EVs group (EV), and EA combined with iPSC-EVs group (EA+EV). EA and EV treatments were performed four times at 0 h, 24 h, 48 h, and 70 h after reperfusion. In the EA group, acupuncture needles (ANDE, Guizhou, China) were inserted into the mice at the “Baihui” acupoint (GV20) and left the “Zusanli” acupoint (ST36) at a depth of 2–3 mm. The stimulation was carried out for 30 min at 2 Hz (intensity, 1 mA) frequency with continuous waves using an electroacupuncture device (KWD-808 II, Great Wall Brand, Baoding, China). Additionally, in EV group, iPSC-EVs were injected into the tail vein of mice, 20 μg per injection in each mouse was performed for a total of four injections. EA combined with iPSC-EVs treatment (EA+EV) utilized the same EA and EV treatment methods described above.

### 2.5 Neurological score

Neurological function and deficits were evaluated 72 h after reperfusion by the Clark score, which is closely related to cerebral infarction. Using this test, the general functional impairment score (0-28 points) and focal functional impairment score (0-28 points) were applied to evaluate the injury of mice. The lowest number of points (0) indicated normal function, and the highest score (28 points) represented the most severe level of functional impairment.

### 2.6 Histopathology staining

For histopathological staining (72 h postreperfusion), frozen brain sections (12 μm thick) were used for Nissl staining, and colon tissues were embedded in paraffin (4 μm thick) with hematoxylin and eosin (H&E) to reveal histopathological lesions. Images of the Nissl staining and H&E staining results were captured using a fluorescence microscope (Carl Zeiss, Germany).

### 2.7 Western blot

Mouse brain tissue and colon tissue were collected from each group. Protein samples were separated on SDS−PAGE gels then electrotransferred to nitrocellulose membranes (Millipore, Burlington, MA, USA). The primary antibodies were hybridized with membranes. The primary antibodies included anti-IL-10 (1:200, ab9969, Abcam, Cambridge, MA, USA) and anti-IL-17 (1:200, ab79056, Abcam, Cambridge, MA, USA). The secondary antibodies incubated with membranes. The ImageJ software were used to scan and analyze protein bands. The bands of brain tissue were normalized to β-tubulin (ab 7291, Abcam, Cambridge, MA, USA), and colon tissue were normalized to β-actin (ab8227, Abcam, Cambridge, MA, USA).

### 2.8 16S rRNA gene sequencing

The contents of the colon were harvested after 72 h, immediately frozen in liquid nitrogen and stored at -80°C. Total genomic DNA samples were extracted using the OMEGA Soil DNA Kit (M5635-02) (Omega Bio-Tek, Norcross, GA, USA). The V3-V4 region of the bacterial 16S rRNA gene was amplified by PCR. The Illumina MiSeq/NovaSeq platform was used to perform double-end sequencing of community DNA fragments. Microbiome bioinformatics was performed with QIIME 2 2019.4 with slight modification according to the official tutorials. Sequence data analyses were mainly performed using QIIME2 and R packages (v3.2.0).

### 2.9 Statistical analysis

The data were analyzed with SPSS 24.0 and were presented as the means ± standard deviations (SD). Comparisons among groups involved the use of one-way analysis of variance (ANOVA) and the Tukey–Kramer *post hoc* multiple comparisons test. Differences among groups were considered significant when the *P* value was < 0.05.

## 3 Results

### 3.1 EA and iPSC-EVs treatments alleviated injury to the brain and intestine after cerebral ischemia−reperfusion

**Figure 1:**
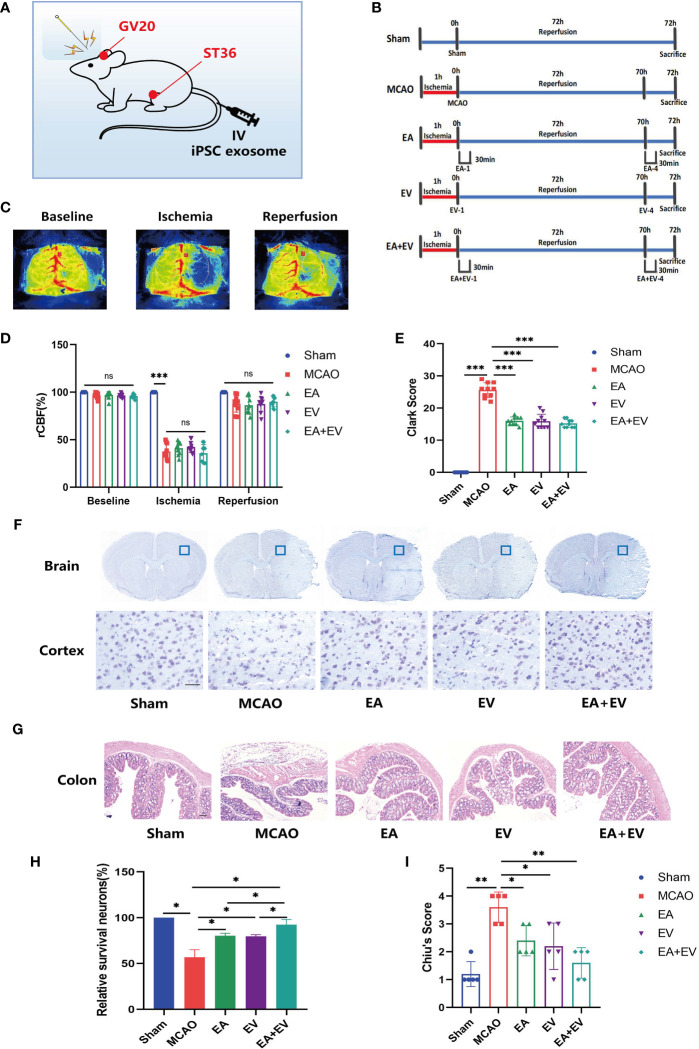
EA and iPSC-EVs treatments reduce brain tissue and intestinal injury after cerebral ischemia−reperfusion. **(A)** Image of mouse EA and iPSC-EVs treatments. **(B)** Diagram of the experimental paradigm used in each group. **(C, D)** Cerebral blood flow was monitored by rCBF laser speckle before and after MCAO and after reperfusion. Scale *** indicates significant differences between the Sham and MCAO groups, ns indicates no differences in MCAO, EA, EV, and EA+EV between groups after ischemia, n=10/group. **(E)** The Clark score was used to evaluate the degree of neurological impairment. **(F)** Representative images of Nissl staining. Scale bar = 50 μm. **(G)** Injury of the colon after cerebral injury. Scale bar = 100 μm. **(H)** Quantification of the number of neurons positive for Nissl staining, n = 4/group. **(I)** Chiu’s score of colon injury after cerebral ischemia. Data were expressed as the mean ± SD, *P < 0.05, **P < 0.01, ***P < 0.001.

To determine the effects of EA and iPSC-EVs on mice subjected to cerebral ischemia−reperfusion injury, a mouse MCAO model was established according to the procedure (29). The acupoints and iPSC-EVs injection points were shown in **Figure 1A**. The mice were randomly divided into five groups, including the Sham operation group (sham), model group (MCAO), electroacupuncture group (EA), iPSC-EVs group (EV), and EA combined with iPSC-EVs group (EA+EV) (**Figure 1B**). Laser speckle cerebral blood flow was monitored before the operation, during the operation, and after reperfusion to ensure successful model establishment (**Figure 1C**). Cerebral blood flow in the MCAO, EA, EV, and EA+EV groups was significantly lower than that in the Sham group (*P* < 0.001, **Figure 1D**). There was no significant difference among the MCAO, EA, EV, and EA+EV groups, and there was no difference in cerebral blood flow after recovery (**Figure 1D**). The Clark neurological function score was determined 72 h after EA and EV treatments (**Figure 1E**). The score in the MCAO group was significantly higher than that in the Sham group (*P* < 0.001, **Figure 1E**). The Clark scores were decreased significantly after EA, EV, and EA+EV treatment 4 times compared with those of the MCAO group (*P* < 0.001, **Figure 1E**). However, there was no statistical significance among the EA, EV, and EA+EV groups (**Figure 1E**). Moreover, Nissl staining showed that unlike in the Sham group, the structure of the cortex on the injured side in MCAO mice was incomplete, the number of Nissl bodies was reduced or they were destroyed, and the arrangement was irregular (*P* < 0.05, **Figures 1F, H**). EA, EV, and EA+EV significantly alleviated neuronal injury (*P* < 0.05, **Figures 1F, H**). There was no significant difference between the EA and EV groups, but significant differences were observed between the EA and EA+EV groups and the EV and EA+EV groups (*P* < 0.05, **Figures 1F, H**). EA, EV, and EA+EV reduced injury after cerebral ischemia−reperfusion. Intriguingly, H&E staining showed that unlike in the Sham group, according to the Chiu’s score the colon tissue in the MCAO group showed obvious pathological changes (*P*<0.01, **Figures 1G, I**), including intestinal mucosal edema, integrity destruction, villi damage, breakage and stripping, and a reduction in the number of goblet cells. The EA, EV, and EA+EV treatment groups exhibited significantly fewer pathological changes of intestinal mucosal edema, villus damage, and goblet cell depletion in colon tissue than the MCAO group (*P* < 0.05, *P* < 0.05, *P* < 0.01, **Figures 1G, I**).

### 3.2 EA and iPSC-EVs treatments regulated the levels of IL-17 and IL-10 in brain and colon tissue after cerebral ischemia−reperfusion

**Figure 2:**
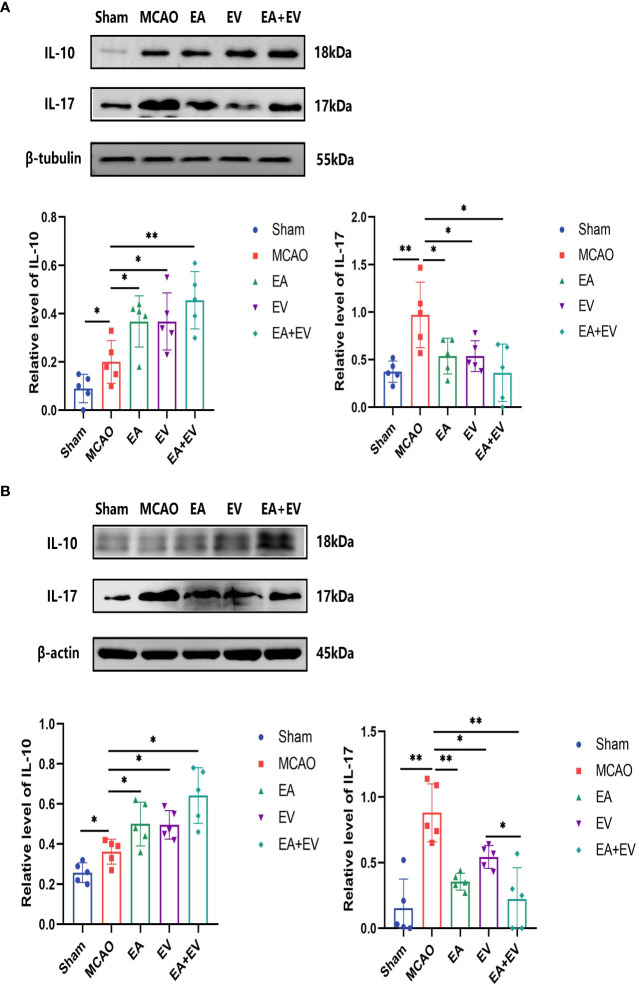
EA and iPSC-EVs treatments regulated the levels of IL-17 and IL-10 in brain and colon tissues after cerebral ischemia reperfusion. **(A)** The levels of IL-17 and IL-10 in brain tissues were measured by western blot, n = 5/group. **(B)** The levels of IL-17 and IL-10 in the colon tissues were measured by western blot, n = 5/group. Data were expressed as the mean ± SD, *P < 0.05, **P < 0.01.

To confirm the protective effects of EA and iPSC-EVs in the brain and colon tissues in MCAO mice, the expression levels of the inflammatory cytokines IL-17 and IL-10 on the brain and colon tissues were measured by western blot. The intestinal flora was dysregulated after cerebral ischemia. IL-17 and IL-10, which were proinflammatory and anti-inflammatory factors, respectively, played an important role in intestinal injury and brain injury. In the brain tissue on the side of the injury, IL-17 expression was significantly increased in the MCAO group (*P <* 0.05, **Figure 2A**) and was significantly decreased in the EA, EV, and EA+EV groups (*P <* 0.05, **Figure 2A**). IL-10 expression increased in the MCAO group (*P <* 0.05, **Figure 2A**) and significantly increased after EA, EV and EA+EV treatments (*P <* 0.05, *P <* 0.05, *P <* 0.01, **Figure 2A**), and EA+EV treatment was more effective than EA and iPSC-EVs treatments alone.

Similarly, in colonic tissue, IL-17 expression was significantly increased in the MCAO group (*P <* 0.05, **Figure 2B**) and significantly decreased in the EA, EV and EA+EV groups (*P <* 0.01, *P <* 0.05 and *P <* 0.01, **Figure 2B**). IL-10 levels increased in the MCAO group (*P <* 0.05, **Figure 2B**) and were significantly increased after EA, EV and EA+EV treatments (*P <* 0.05, **Figure 2B**). Together, these results suggested that EA, EV, and EA+EV treatments regulated inflammatory factors to exert a protective effect on the brain and intestinal tissue, and EA+EV treatment displayed a more obvious effect.

### 3.3 EA and iPSC-EVs treatments modulated the microbiota composition in the intestines of the mice after cerebral ischemia−reperfusion

**Figure 3:**
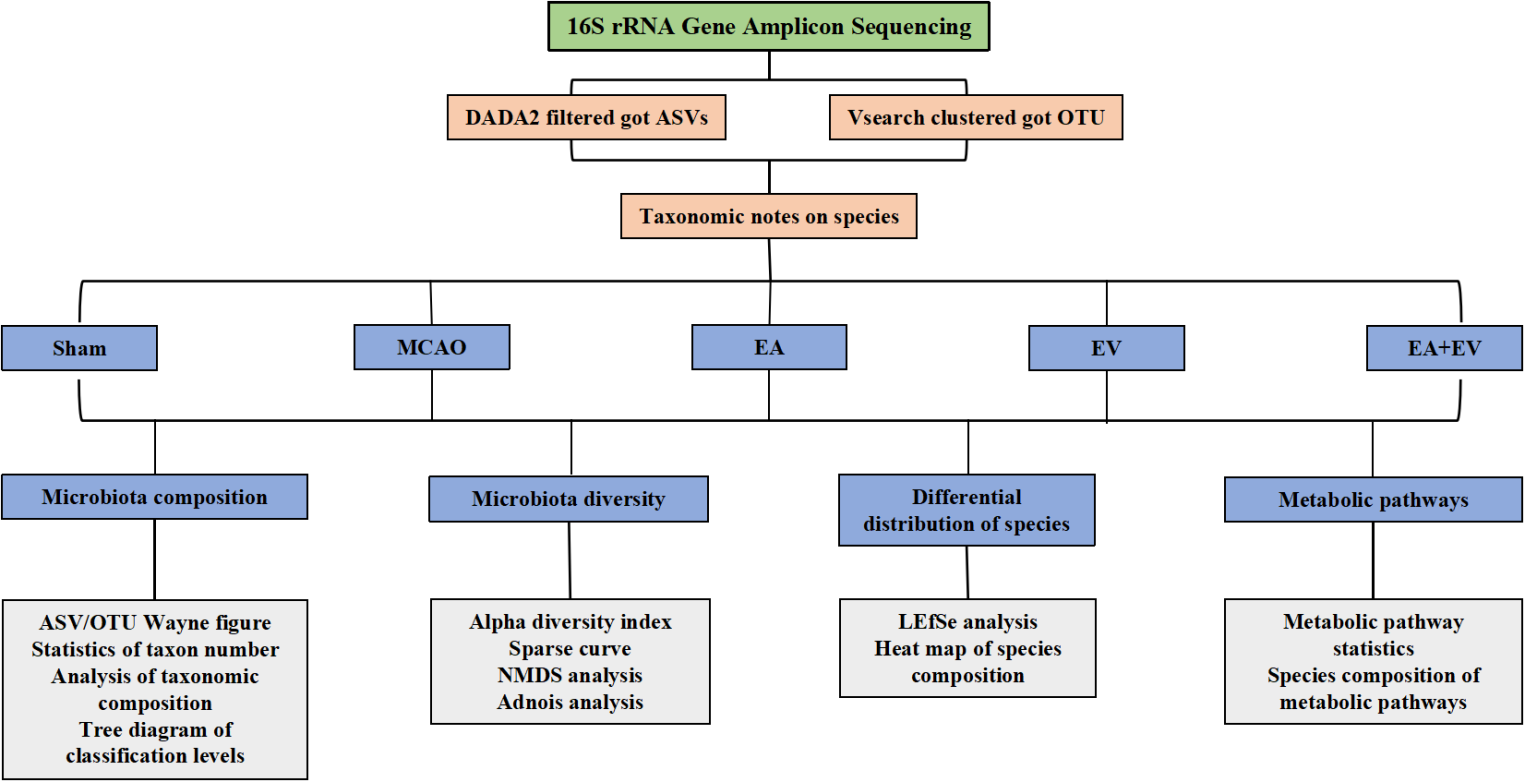
16S rRNA gene amplicon sequencing bioinformatics and statistical analysis.

To further explore the possible protective mechanism of EA, EV, and EA+EV treatments, we performed stool 16S rRNA gene sequencing analysis to distinguish between the intestinal microbiota composition in normal mice and MCAO mice. QIIME 2 2019.4 software was applied for microbiome bioinformatics with slight modification according to the official tutorials. Sequence data analyses were mainly performed using QIIME2 and R packages (v3.2.0) as shown in **Figure 3**.

**Figure 4:**
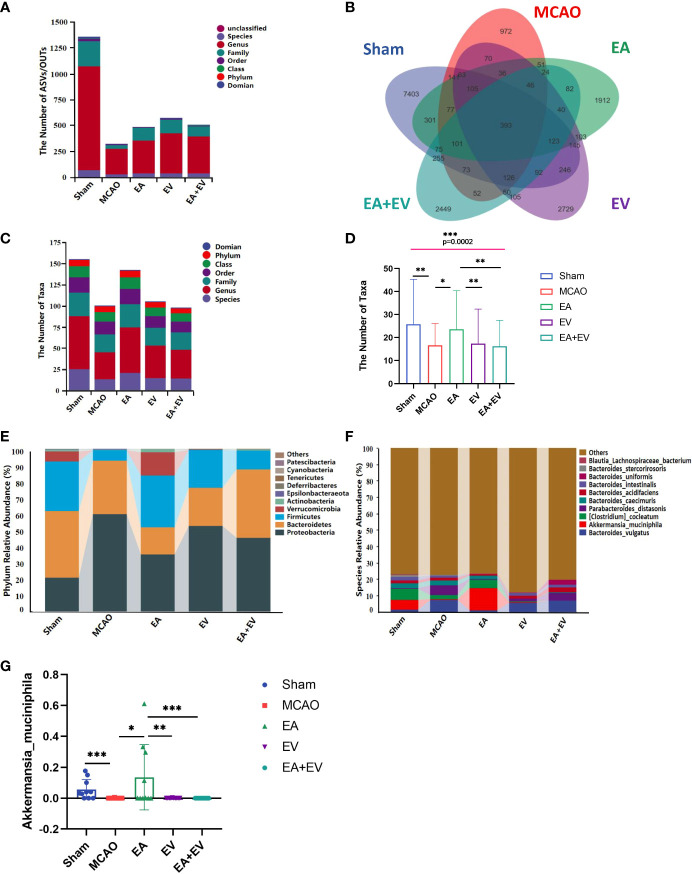
EA and iPSC-EVs treatments modulated microbiota composition after cerebral ischemia−reperfusion. **(A)** The ASV/OTU number at each taxonomic level of phylum, class, order, family, genus, and species. The process used for species annotation involves comparing the data with a reference sequence database and scoring the results. **(B)** ASV/OTU Wayne diagram. To determine which species were common and which were unique among different samples (groups), ASV/OTU abundance tables were used to generate a Venn diagram. **(C, D)** Species composition taxon number statistics. **(E, F)** Taxonomic composition statistics at the phylum and species levels. **(G)** Akkermansia muciniphila flora abundance, n= 10/group, data wre expressed as the mean ± SD, *P < 0.05, **P < 0.01, and ***P < 0.001.

Regarding the number of microorganisms, the abundance of intestinal microbial species in the MCAO group was decreased significantly compared with that in the Sham group. EA, EV, and EA+EV treatments restored the abundance of intestinal microbial species (**Figures 4A, B**). It was very interesting that the number of taxonomic units in the MCAO group was significantly lower than that in the Sham group. EA treatment significantly improved the number of taxonomic units in the intestinal microbiota (*P <* 0.05, **Figures 4C, D**), and the effect was more significant in this group than in the EV and EA+EV groups (*P <*0.01, **Figures 4C, D**; **Figure S1**). Based on the statistics obtained from the flattened amplicon sequence variant (ASV)/operating taxonomic units (OTU) table, the specific taxonomic composition of microbial communities and the number of taxa contained in each sample at each taxonomic level were calculated. The phylum level analysis showed that the abundance of Proteobacteria in the MCAO group was significantly higher than that in the Sham group (**Figure 4E**), indicating the dysregulation of intestinal microorganisms. EA, EV, and EA+EV treatments decreased their levels, and the effect of EA was more significant than those of EV and EA+EV treatments (**Figure 4E**). Bacteroidetes was a phylum involved in metabolic activities in the colon. EA+EV treatment was associated with a high proportion of Bacteroidetes compared with that of the Sham group (**Figure 4E**). Firmicutes and Verrucomicrobia were the most beneficial bacteria, and a high proportion was observed in the Sham and EA groups (**Figure 4E**). In particular, Akkermansia was associated with cardiovascular and cerebrovascular diseases and decreased blood flow after cerebral ischemia, promoting the balance of metabolism in the body (42, 43). The abundance of Akkermansia was significantly increased in the EA group (**Figures 4F, G**). These findings indicate that EA has a more substantial effect on regulating the balance of metabolism. EV and EA+EV treatments had more prominent effects on the overall number of gut microbes, and EA was potentially more involved in regulating the specific taxa and the taxonomic composition.

### 3.4 EA and iPSC-EVs treatments regulated the microbiota diversity in the intestines of mice after cerebral ischemia−reperfusion

The alpha diversity index represents the diversity of species and the beta diversity index represents the diversity of species between habitats to comprehensively evaluate the overall diversity of species. Alpha diversity refers to indexes including the richness, diversity, and evenness of the species in partially uniform habitats and is also known as within-habitat diversity. Here, we used richness (Chao1, Observed Species), diversity (Shannon, Simpson), diversity based on evolution (Faith’s PD), evenness (Pielou’s Evenness) and coverage (Good’s coverage) to comprehensively analyze alpha diversity.

**Figure 5:**
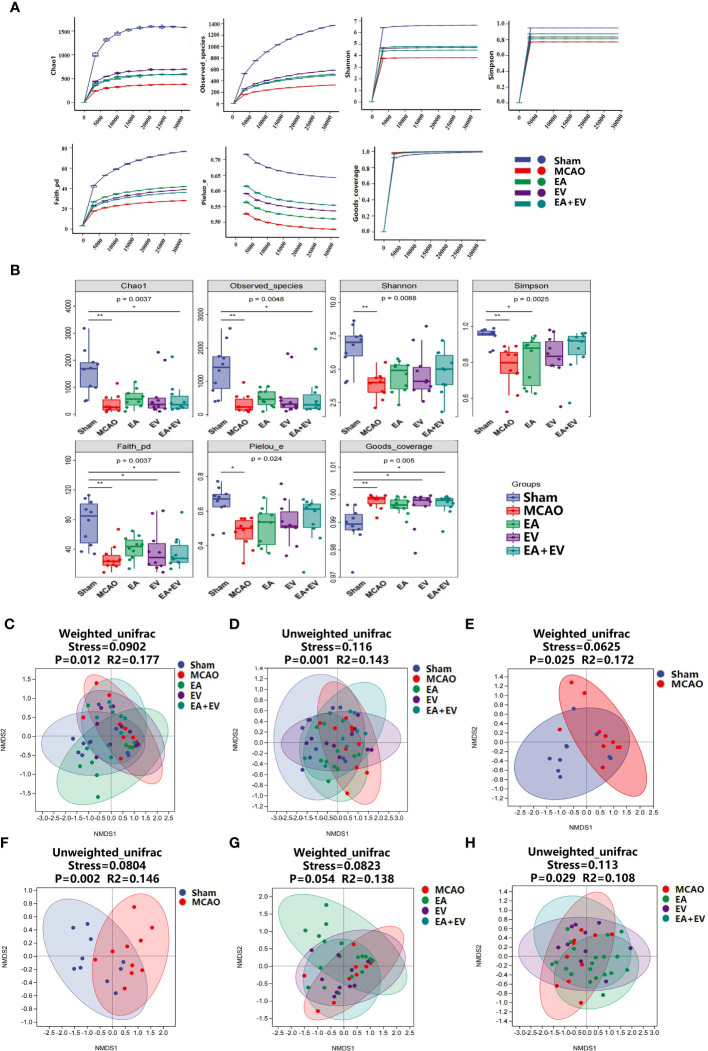
EA and iPSC-EVs treatments regulated the microbiota alpha and beta diversity after cerebral ischemia−reperfusion. **(A, B)** Alpha diversity of microbial communities (Chao1, observed species index for richness; Shannon and Simpson index table characteristic diversity; Faith’s PD index represents diversity based on evolution. Pielou’s evenness index was used to characterize the evenness. Good’s coverage index represents the coverage). **(C–H)** Nonmetric multidimensional scaling analysis (NMDS) of beta diversity in microbial communities (weighted unifrac, unweighted unifrac). NMDS simplifies the data structure by reducing the dimension of the sample distance matrix to describe the distribution characteristics of samples at a specific distance scale. The lower the stress value of the NMDS, the better. The NMDS analysis results are more reliable when the stress value is less than 0.2, n = 10/group, data were expressed as the mean ± SD, *P < 0.05, **P < 0.01.

Regarding the alpha diversity index, there were significant differences between the MCAO and Sham groups in richness and diversity and in diversity based on evolution, evenness, and coverage (*P* <0.05) (**Figures 5A, B**). The Chao1 value and number of observed species (the richness sparse curve) were more significant in the EV group; the Shannon, Simpson’s and Pielou’s evenness (the diversity and evenness) values were more significant in the EA+EV groups; the Faith’s PD (the diversity based on evolution) value was more significant in the EA group; and Good’s coverage index represented coverage with almost no difference (**Figure 5A**).

Beta diversity, also known as between-habitat diversity, refers to the dissimilarity of species composition or the replacement rate of species along environmental gradients among different communities that change along environmental gradients. Using nonmetric multidimensional scaling analysis (NMDS) unconstrained ranking, dimensionality reduction of multidimensional microbial data can be carried out. Finally, the main trends in the data can be observed by analyzing the distribution of samples in the continuous ranking axis.

When the NMDS value is less than 0.2, the results of the NMDS analysis are more reliable. The weighted unifrac (Stress=0.0902, P=0.012, R2=0.177) and unweighted unifrac (Stress=0.116, P=0.001, R2=0.143) results in the Sham, MCAO, EA, EV, and EA+EV groups were statistically significant (**Figures 5C, D**). Sham and MCAO resulted in significantly weighted unifrac (Stress=0.0625, P=0.025, R2=0.172) and unweighted unifrac (Stress=0.0804, P=0.002, R2=0.146) values (**Figures 5E, F**). The stress values in the MCAO and EA, EV, EA+EV groups calculated in weighted unifrac (Stress=0.0823, P=0.054, R2=0.138) and unweighted unifrac (Stress=0.113, P=0.029 R2=0.108) stress assessments were less than 0.2 and were statistically significant (**Figures 5G, H**).

In summary, these results suggest that EA, EV, and EA+EV could improve alpha and beta diversity, especially with EV and EA+EV treatments, which exhibited advantages in the alpha diversity index. Regarding improvements in the beta diversity index, EA was better than EV and EA+EV treatments.

### 3.5 EA and iPSC-EVs treatments regulated the differential distribution of species after cerebral ischemia−reperfusion

After exploring the differences in microbial community composition through assessments of beta diversity, we investigated which species were responsible for these differences. LEfSe analysis was applied for this analysis. To further compare the differences in species composition among samples and show the distribution trend of species abundance in each sample, heatmaps were used for species composition analysis.

**Figure 6:**
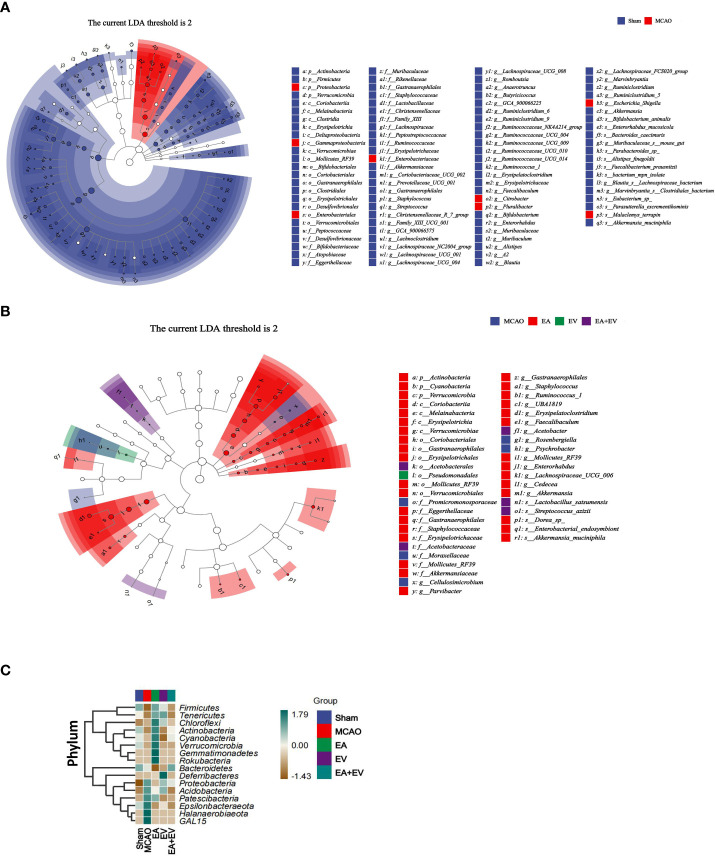
EA and iPSC-EVs treatments can regulate microbiota diversity after cerebral ischemia−reperfusion. **(A)** LEfSe analysis of microbial differences at the species level between the Sham and MCAO groups **(B)** LEfSe analysis of microbial differences at the species level among MCAO, EA, EV, and EA+EV. The taxonomic cladogram shows the taxonomic hierarchical distribution of marker species in each sample. **(C)** Heatmap of species composition at the phylum level, n = 10/group.

The LEfSe method was used to identify the differences between the Sham and MCAO groups (**Figure 6A**) and among the MCAO groups subjected to EA, EV, and EA+EV treatments at different classification levels (**Figure 6B**). Based on the comparisons of the MCAO and Sham groups, Proteobacteria may be a pathogenic bacterium related to the mechanism of postischemic injury (**Figure 6A**). Based on comparisons between the EA, EV, and EA+EV treatment groups, EA improved intestinal microbiota imbalance by affecting Actinobacteria, Coriobacteria, and Verrucomicrobia and EA+EV through Verrucomicrobia (**Figure 6B**). It can be concluded that EA resulted in the most significant improvement in intestinal microbiota (**Figure S2A–E**).

Heatmaps were generated to show the distribution and abundance of each species at the phylum level (**Figure 6C**). To further compare the differences in species composition among samples and show the distribution trend of species abundance in each sample, heatmaps were used for phylum composition analysis. By default, we used abundance data from the top 20 genera with average abundance for heatmapping. The EA group was significant at the phylum level (Firmicutes, Tenericutes, Chloroflexi, Actinobacteria, Cyanobacteria, Verrucomicrobia, Gemmatimonadetes, Rokubacteria, Bacteroidetes). The MCAO group was significant at the phylum level (Proteobacteria, Acidobacteria, Patescibacteria, Epsilonbacteraeota, Halanaerobiaeota, GAL15). The EV group exhibited Deferribacteres at the phylum level (Mucispirillum schaedleri, Parabacteroides goldsteinii). The Sham and EA+EV groups exhibited specific regulation at the phylum level.

### 3.6 EA and iPSC-EVs treatments regulated the metabolic pathways after cerebral ischemia−reperfusion

**Figure 7:**
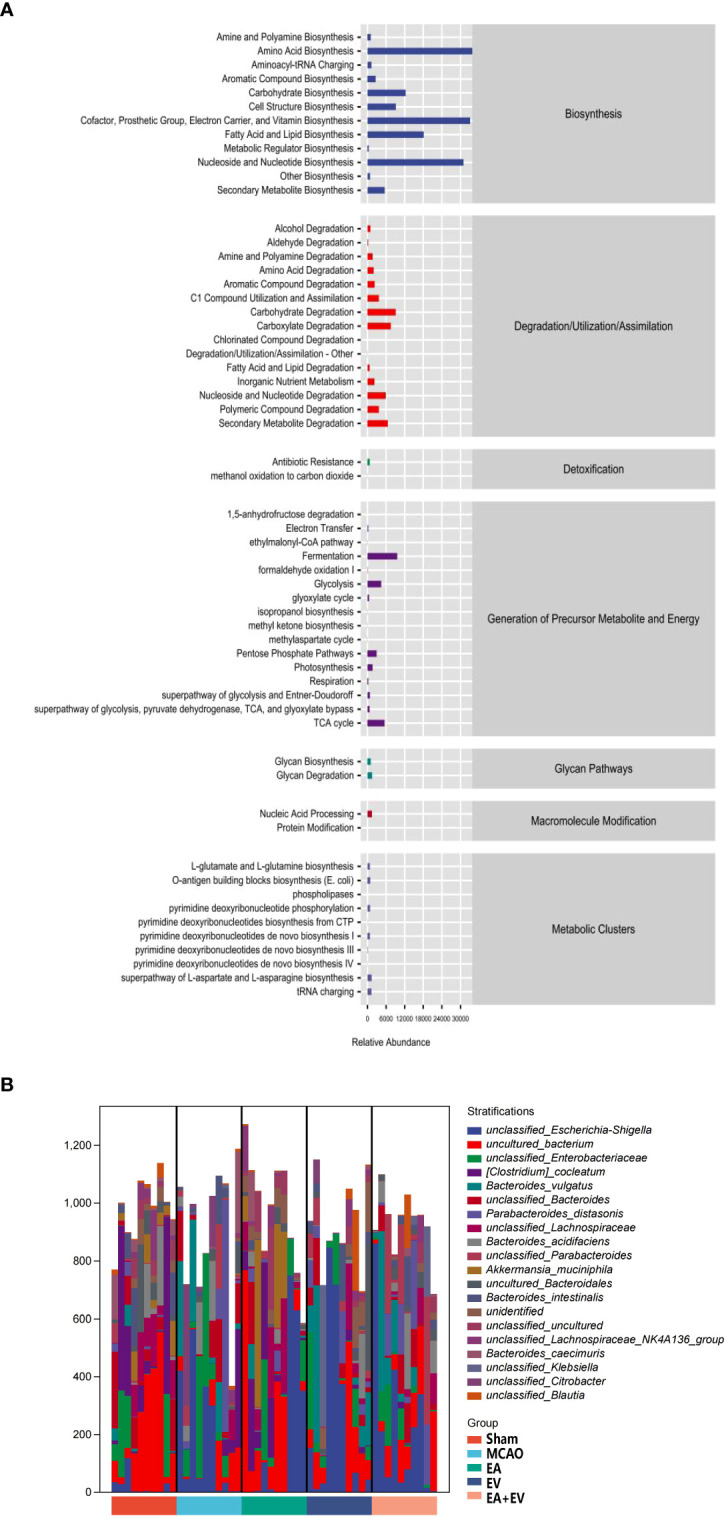
EA and iPSC-EVs treatments may regulate metabolic pathways after cerebral ischemia−reperfusion. **(A)** Metabolic pathway statistics. **(B)** Species composition of metabolic pathways, n= 10/group.

The above analysis focused on the diversity and species composition of the flora. For the study of microbial ecology, we also paid attention to the functional potential of the microbiota. The functional units obtained were used to obtain the abundance value of metabolic pathways according to the metabolic pathway database and certain calculation methods. Metabolic pathways were divided into six broad categories, including metabolism, genetic information processing, environmental information processing, cellular process processes, organismal systems, and human diseases. Each of these was further divided into multiple hierarchies (**Figure 7A**).

Having obtained the functional components of the samples/groups, especially some of the differential pathways, we next investigated which species encoded the genes with these functional potentials. A stratified sample metabolic pathway abundance table was generated for species composition pathway analysis (**Figure 7B**). It was confirmed that all or most of the functions involved in metabolic pathways could be independently performed by a certain bacterial species.

## 4 Discussion

Stroke promotes an increase in the local inflammatory response, resulting in two-way communication and immune signal exchange between the gut and brain through the brain-gut axis. After cerebral ischemia, intestinal microbiota dysregulation produces an inflammatory response. In the current study, we investigated the protective effects of EA and iPSC-EVs on brain and colon tissues and the regulatory effects on intestinal microbiota using a mouse model of MCAO. EA, iPSC-EVs, and EA combined with iPSC-EVs treatments reduced damage to brain and colon tissue, and their protective effect was related to the regulation of intestinal microorganisms.

This study showed that EA, iPSC-EVs and EA combined with iPSC-EVs increased the number of neurons (Nissl staining), improved neural function (Clark score), alleviated inflammation in MCAO mice, reduced colon injury, inhibited inflammation, and regulated gastrointestinal microbes. Regarding the inflammatory response, the effect of EA combined with iPSC-EVs treatment was more significant than the effects of EA and iPSC-EVs. Regarding intestinal microbiota regulation, EA more significantly regulated beneficial flora, and iPSC-EVs and EA combined with iPSC-EVs treatments more significantly regulated microbial diversity. We believe that the combination of EA and iPSC-EVs may have synergistic neuroprotective effects that are greater than those of the use of iPSC-EVs or EA alone, which is consistent with previous studies (29).

Acupuncture treatment, as an effective method of traditional Chinese medicine for stroke treatment, has unique advantages, especially with the correct choice of points. GV20 is located in the head and has the effect of awakening the brain and enlightening the body, which is a point of the governor meridian involving the governor meridian veins in the brain, for the treatment of the encephalopathy effect point. ST36 belongs to the Stomach Meridian of Foot-Yangming, which is targeted to treat gastrointestinal diseases, paralysis of lower limbs, mental disease and deficiency of fatigue. According to the literature reports and previous studies, GV20 and ST36 can reduce brain edema, inhibit inflammation, improve mitochondrial function and promote nerve regeneration (37, 38, 44–46). EA and iPSC-EVs treatments promote the Treg response and regulate Th17 and Th1 inflammatory responses to protect the brain (29).

Acupuncture plays a unique role in regulating gastrointestinal inflammation and intestinal microbiota. Acupuncture at ST36 can inhibit neuroinflammation and intestinal microbiota dysregulation in a mouse model of Parkinson’s disease (47, 48), preserves intestinal barrier integrity by modulating the gut microbiota in dextran sulfate sodium (DSS)-induced chronic colitis (49), and may ameliorate spinal cord injury (SCI) by modulating microbiota and metabolites (50). Relevant studies have shown that acupuncture has a positive effect on the treatment of ischemic stroke through the interaction between the gut microbiota and the immune response; it can correct disorders in intestinal microbiota, reduce the immune inflammatory response, and promote the recovery of brain tissue damage and neurological function after stroke (51, 52). Acupuncture at LI11, GB34 and ST36 promotes recovery after stroke by regulating intestinal microecology and plasma metabolism (53). Acupuncture at GV20 and ST36 can benignly modulate gut microbiota dysbiosis and significantly reduce intestinal inflammation (54).

Exosomes affect inflammatory bowel disease (IBD)-related pathways, such as immune responses, barrier function, and gut microbiota (55). Exosomes from different sources and the components of exosomes have different regulatory effects on intestinal immune regulation and microbiota (55, 56). Additionally, exosomes intervene in the intestinal inflammation caused by the imbalance of intestinal flora after stroke and play a neuroprotective role through the brain-gut axis (57).

Innate immune cells respond within hours after ischemia and then mount an adaptive immune response by activating T and B lymphocytes. T-cell subsets can help or aggravate ischemic brain injury (58). Proinflammatory Th1, Th17, and γδT cells are commonly associated with increased inflammatory damage, while regulatory T cells are known to inhibit postischemic inflammation mediated by IL-17 by increasing the secretion of the anti-inflammatory cytokine IL-10 (58–60). Our research showed that EA, iPSC-EVs, and EA combined with iPSC-EVs treatments significantly decreased IL-17 levels and increased IL-10 levels in the brain and colonic tissue, and EA combined with iPSC-EVs treatment was more effective than EA or iPSC-EVs treatment alone. EA, iPSC-EVs, and EA combined with iPSC-EVs treatments can regulate inflammatory factors to exert a protective effect on the brain and intestinal tissue, and EA combined with iPSC-EVs treatment has a more obvious effect. This result was consistent with previous literature studies (61).

The intestinal microbiota is dysregulated after cerebral ischemia, which causes inflammation; IL-17 expression increases, and inflammation further aggravates the dysbiosis of intestinal flora; decreasing IL-17 and increasing IL-10 levels may regulate the intestinal microbiota, which may be a mechanism that inhibits the transition into an inflammatory state. This effect may be due to Firmicutes, Bacteroidetes, and actinobacteria, which exhibit severely reduced abundance after stroke (17). An imbalance in gut microbiota can contribute to stroke and may exacerbate its effects. The prognosis of stroke can be improved with a variety of treatments. A large number of gut microbes and multiple metabolites may alter the impact of the gut microbiome on stroke outcomes. The brain-gut axis is a neuro-immune network that is bidirectional to the gut. Changes in the cerebral and intestinal axis can also cause changes in the structure of the microflora. The dysfunctional intestinal microflora can stimulate inflammatory response, change the intestinal mucosal permeability, affect the inflammatory factors to aggravate the cerebral ischemia injury, and then feed back to the gut and microflora, and the cycle repeats. Acupuncture and moxibustion can regulate the various links of the cerebral and intestinal axis and block this vicious cycle (62, 63).

Regarding the gut microbiome, iPSC-EVs and EA combined with iPSC-EVs treatments predominantly regulate the overall number, abundance, diversity of gut microbes. At the phylum level, increased Proteobacteria abundance is a microbiological characteristic of intestinal flora dysregulation. After cerebral ischemia, its abundance increased significantly in the MCAO group, and EA, iPSC-EVs, and EA combined with iPSC-EVs treatments reduced it.The abundance of Firmicutes and Verrucomicrobia, which are beneficial bacteria, can be increased by EA. Furthermore, Akkermansia abundance was increased by EA, which protected the integrity of intestinal epithelial cells and the mucus layer and played a protective metabolic role. Akkermansia is associated with cardiovascular and cerebrovascular diseases and decreases in blood flow after cerebral ischemia, promoting the balance of metabolism in the body. Bacteroidetes abundance can be increased by EA combined with iPSC-EVs treatment, and Bacteroidetes are involved in many important metabolic activities in the colon. We speculate that EA and iPSC-EVs treatments have different regulatory mechanisms involving gut microbes. In this study, we used ST36, which is the “He-sea Point” and “lower He-sea Point” of the Stomach Meridian of Foot-Yangming and has the effect of strengthening the spleen and stomach, as stated in the expressions “the belly ST36 stay” and “He treats the six Fu-Organs”. This point is used for treating stomach pain, vomiting, hiccups, abdominal distension, abdominal pain, bowel cramping, indigestion, diarrhea, constipation, etc. Based on the characteristics of meridians and the acupoints to which they belong, treating these regions has a unique regulatory effect on intestinal microorganisms. The iPSC-EVs maybe influence intestinal microbes by improving inflammatory responses (64).

The alpha and beta diversity of the microbiota is positively correlated with health. The more abundant different microorganisms in the gut, the more positively correlated with metabolic health (65, 66). The imbalance of intestinal microbiota is generally accompanied by a decrease in the alpha diversity of the microbiota (67, 68). Short-term intervention did not increase the abundance of intestinal microbiota but increased beneficial bacteria, leading to an increase in β diversity before and after intervention (69).

EA, iPSC-EVs, and EA combined with iPSC-EVs treatments improved alpha and beta diversity. Regarding improvements in the alpha diversity index, the iPSC-EVs and EA combined with iPSC-EVs treatments had advantages; regarding the beta diversity index, EA was better than the iPSC-EVs and EA combined with iPSC-EVs treatments. LEfSe also showed that EA improved the intestinal microbiota imbalance by affecting Actinobacteria, Cyanobacteria, Verrucomicrobia, and EA combined with iPSC-EVs through Verrucomicrobia. Additionally, the heatmap showed that the EA combined with iPSC-EVs group was comparable to the Sham group at the phylum level. Overall, the efficacy of EA combined with iPSC-EVs treatment was more significant than that of EA or iPSC-EVs treatments alone. The gut bacteria can also participate in the immune response and in neuroinflammation through microbiota-derived metabolites (70), and the results of this study also demonstrated the metabolic pathways that may be affected by gut microbiota. Acupuncture treatment has a positive effect on neurological diseases by regulating immune responses and changing the metabolism of related tissues (71). The specific metabolic pathways and the mechanisms of metabolite production need to be further studied.

This study showed that iPSC-EVs used for the regulation of intestinal flora species were better at targeting the overall abundance of specific species; the adjustment of beneficial bacteria was not very substantial, and previous reports suggest that mesenchymal origin cells secrete iPSC-EVs and that those secreted by intestinal epithelial cells (IECs) have a significant role (72, 73). Regarding the inhibition of the inflammatory response, this study was consistent with previous literature (74). The mechanism through which iPSC-EVs regulate the intestinal flora needs to be further studied.

Electroacupuncture has been widely used to treat ischemic stroke, especially in the sequelae of stroke. Recently, iPSC-EVs have become one of the focuses in the research field because of their powerful ability to inhibit inflammatory response and to promote tissue regeneration. In addition, iPSC-EVs exhibit good safety and the capacity to pass through the blood-brain barrier while avoiding tumorigenicity of iPSCs. We believe that the combination of acupuncture and iPSC-EVs would be a safe and efficient treatment strategy for ischemic stroke in clinic. Next, we will focus to develop iPSC-EVs as a drug and launch a clinical study with this combination strategy.

## 5 Conclusion

Electroacupuncture and iPSC-EVs treatments significantly improved neurological function and neuronal and intestinal tract injury, regulated the intestinal immunity as the levels of IL-17 and IL-10 in brain and colon tissue through MGBA and modulated the microbiota composition and diversity as well as the differential distribution of species in the intestines of the mice after cerebral ischemia−reperfusion. EA and iPSC-EVs treatments positively impacting the outcomes of ischemic stroke and provided new insights into the application of EA combined with iPSC-EVs as a treatment for ischemic stroke.

## Data availability statement

The datasets presented in this study can be found in online repositories. The names of the repository/repositories and accession number(s) can be found below: PRJNA906348 (SRA).

## Ethics statement

The animal study was reviewed and approved by the National Institutes for Animal Research and approved by the Ethics Committee for Animal Experimentation Peking Union Medical College Hospital of the Chinese Academy of Medical Sciences.

## Author contributions

QZ, HS and JZ planned and designed the experiments. QZ, PD, SC, HX and YZ coordinated all the experiments. QZ performed behavioral experiments, Histopathology Staining, and Western Blot. QZ, PD, SC, HX and YZ assisted surgery and behavioral experiments. QZ carried out imaging experiment. QZ provided knowledge on 16s rRNA sequence data analysis. QZ analyzed the data and presented the figures. QZ, SC, and HX drafted the manuscript. QZ, HC, HS and JZ reviewed and revised the manuscript. All authors contributed to the article and approved the submitted version.
