# Supplementary material for: Electroacupuncture and human iPSC-derived small extracellular vesicles regulate the gut microbiota in ischemic stroke via the brain-gut axis
Source: Front Immunol. 2023 Jan 20;14:1107559. doi: 10.3389/fimmu.2023.1107559 (PMC9895941; doi:10.3389/fimmu.2023.1107559)
Supplement: Supplementary file 1 [file DataSheet_1.pdf]

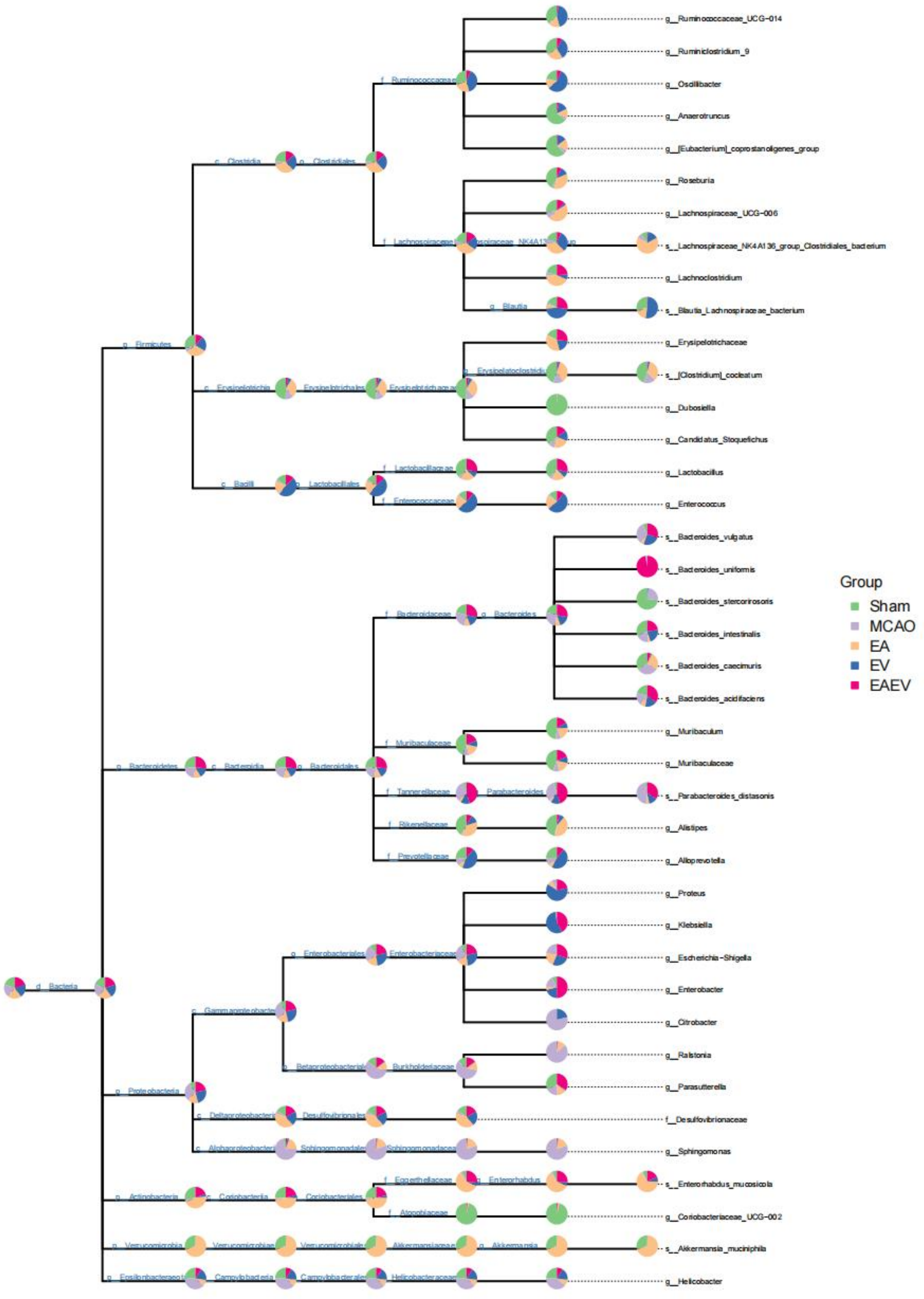

Figure S1. Taxonomic rank tree of species using abundance information. n = 10/group. Hierarchical dendrograms were used to show the taxonomic composition of microbial communities at each level, and pie charts were used to show the composition proportions of different taxonomic units in different.

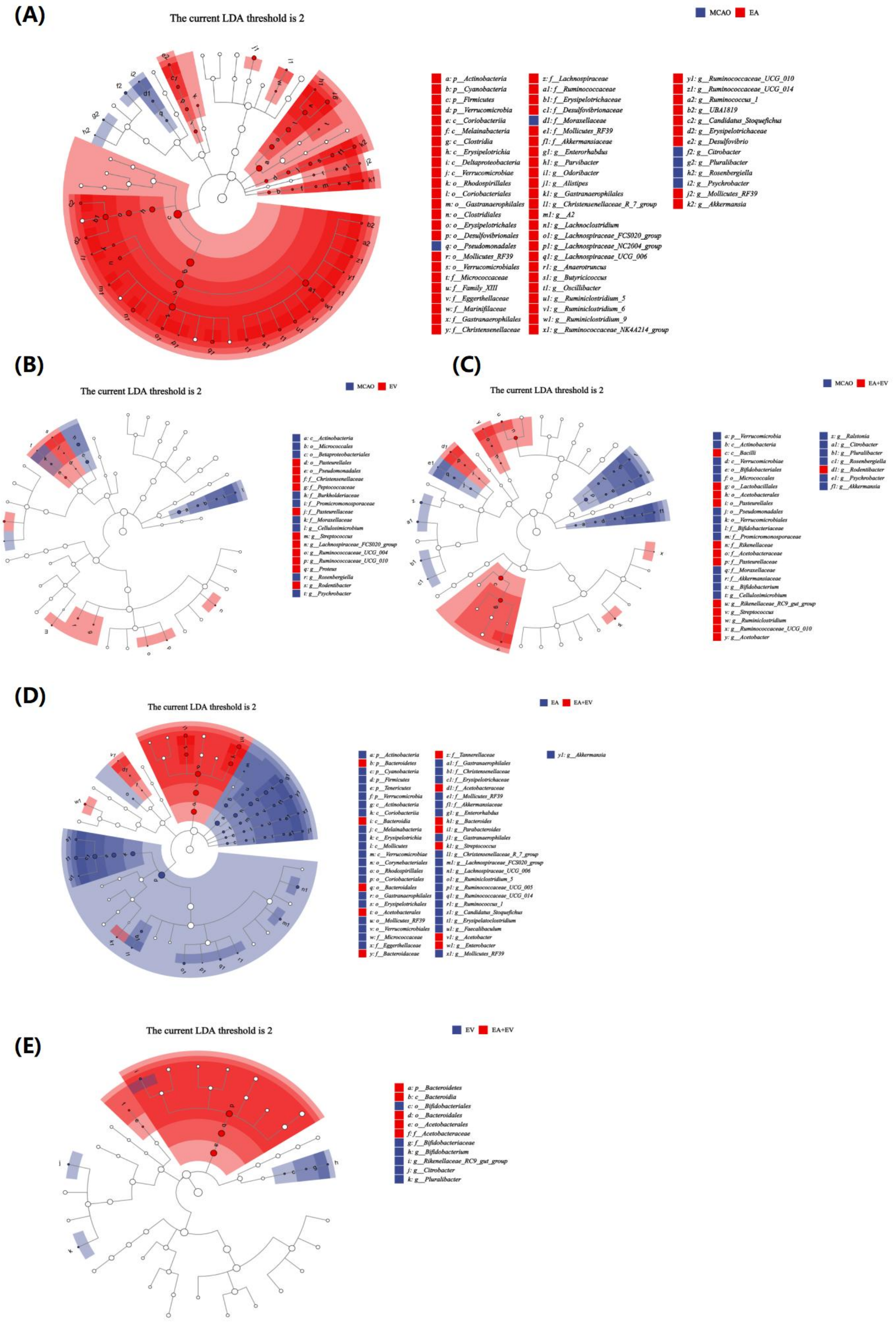

Figure S2. LEfSe analysis of microbial differences at the species level between MCAO and EA (A), MCAO and EV (B), MCAO, EA+EV (C), EA and EV (D), EV and EA+EV (E).
